# The digital biomarker discovery pipeline: An open-source software platform for the development of digital biomarkers using mHealth and wearables data

**DOI:** 10.1017/cts.2020.511

**Published:** 2020-07-14

**Authors:** Brinnae Bent, Ke Wang, Emilia Grzesiak, Chentian Jiang, Yuankai Qi, Yihang Jiang, Peter Cho, Kyle Zingler, Felix Ikponmwosa Ogbeide, Arthur Zhao, Ryan Runge, Ida Sim, Jessilyn Dunn

**Affiliations:** 1 Department of Biomedical Engineering, Duke University, Durham, NC, USA; 2 School of Medicine, Stanford University, Palo Alto, CA, USA; 3 Department of Medicine, University of California, San Francisco, CA, USA; 4 Department of Bioinformatics and Biostatistics, Duke University, Durham, NC, USA

**Keywords:** Biomedical informatics, wearable sensors, health information management, open source, digital medicine, digital health, mHealth

## Abstract

**Introduction:**

Digital health is rapidly expanding due to surging healthcare costs, deteriorating health outcomes, and the growing prevalence and accessibility of mobile health (mHealth) and wearable technology. Data from Biometric Monitoring Technologies (BioMeTs), including mHealth and wearables, can be transformed into *digital biomarkers* that act as indicators of health outcomes and can be used to diagnose and monitor a number of chronic diseases and conditions. There are many challenges faced by digital biomarker development, including a lack of regulatory oversight, limited funding opportunities, general mistrust of sharing personal data, and a shortage of open-source data and code. Further, the process of transforming data into digital biomarkers is computationally expensive, and standards and validation methods in digital biomarker research are lacking.

**Methods:**

In order to provide a collaborative, standardized space for digital biomarker research and validation, we present the first comprehensive, open-source software platform for end-to-end digital biomarker development: *The Digital Biomarker Discovery Pipeline (DBDP)*.

**Results:**

Here, we detail the general DBDP framework as well as three robust modules within the DBDP that have been developed for specific digital biomarker discovery use cases.

**Conclusions:**

The clear need for such a platform will accelerate the DBDP’s adoption as the industry standard for digital biomarker development and will support its role as the epicenter of digital biomarker collaboration and exploration.

## Introduction

The digital health landscape has seen rapidly expanding growth due to the number of chronically ill patients and health system utilization in the USA at an all-time high [1]. Mobile devices and wearables, otherwise known as Biometric Monitoring Technologies (BioMeTs), have facilitated continuous monitoring beyond clinic visits and have enabled significant developments in personalized medicine and mobile health (mHealth) [2,3]. Digital biomarkers are digitally collected data from BioMeTs (e.g., glucose levels) from a continuous glucose monitor (CGM) that are transformed into indicators of health outcomes (e.g., diabetic state). They can be used to provide biomedical insights or improve health decision-making (e.g., encourage healthy lifestyle changes). Research in digital biomarker development spans fields and disease states, from movement-related disorders [4] to breast cancer [5] to Alzheimer’s disease [6], and can conceivably be applied to any area of health, wellness, and medicine. In the past decade, the number of digital biomarker studies indexed in PubMed has increased by 325% (Supplementary Fig. 1). Because only 13% of all research articles published are open access, and even fewer provide their code and/or data, there is a critical need to build an open-source community for digital biomarker development [7]. Currently, digital biomarker development processes are siloed, resulting in numerous studies with digital biomarkers that are not validated properly [8] or are duplicates of already existing digital biomarkers. Open-source digital biomarker development is necessary to broaden the validation of digital biomarkers, reduce duplication, and expedite innovation.

**Fig. 1.**
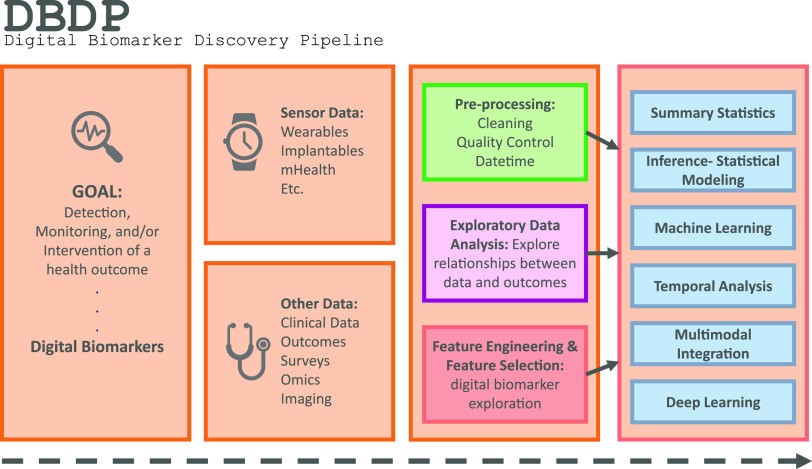
The DBDP.

Mobile and wearable devices continue to gain popularity. Currently, 81% of Americans and 45% of the global population have a smartphone [9,10]. Given the ubiquity of smartphones, mHealth is expected to reach a market size of $236B by 2026 [11]. There are over 300,000 mHealth apps available on the major platforms, and over 60% of people with smartphones have downloaded at least one mHealth app [12]. The popularity of wearable devices is also at an all-time high: by 2021, it is expected that 121 million Americans will use wearable devices [13]. The accessibility of mobile and wearable technology affords an unprecedented opportunity to provide mHealth care globally, and particularly to populations with limited healthcare accessibility, including low-income and rural populations that stand to benefit most from mHealth. mHealth monitoring and interventions are promising because they can improve the health monitoring of patients who are unable to make frequent visits to a health care facility.

The combined market growth in healthcare and mobile and wearable technologies has prompted BioMeT manufacturers to develop algorithms to process raw sensor data and aggregate this data into various health-related metrics. For example, while most wearable BioMeT manufacturers do not provide sample-level sensor accelerometry data, they instead provide aggregate metrics like “activity intensity/duration” or “step count.” For example, Apple recently developed an Food and Drug Administration-cleared algorithm for binary (yes/no) detection of atrial fibrillation using the Apple Watch wrist-based electrocardiogram (ECG) [14]. These aggregate metrics may themselves act as digital biomarkers, or the metrics may be used and combined by researchers and clinicians to develop composite digital biomarkers. One of the major challenges faced by the research and medical communities is that the manufacturer-developed algorithms are nearly always proprietary and information about the verification and validation process of BioMeTs and digital biomarkers is not released to the public [8]. For robust and reproducible digital biomarkers, openness and transparency surrounding the evaluation of these digital tools is critical [8,15–18].

mHealth and wearables data present unique bioinformatic challenges. Providing open-source tools that can be validated by the digital biomarker community would not only make discovering digital biomarkers more accessible but would also instill confidence in their translation into clinical and research settings. Open resources that bridge the stages of digital biomarker development will also enable those with different skill sets (i.e., computational expertise or clinical domain knowledge) to collaborate toward new digital biomarker discovery. Further, tools that allow for collaboration in improving algorithms, validating known digital biomarkers, and discovering new digital biomarkers will enable much-needed standardization and interoperability in this space.

In the past two decades, the field of high-throughput biomolecular analysis set a focus on developing data standards and open platforms for sharing code and data and validating bioinformatic pipelines, which accelerated the pace of research in the genomics community [19,20] and cultivated research in this area [19,21–23]. Similarly, we aim to develop a standardized, open-source data and software platform for the field of digital biomarkers that will facilitate rapid research progress and collaboration.

To address the need for an open resource of computational digital biomarker development tools, here, we present the *Digital Biomarker Discovery Pipeline (DBDP)*, an open-source software to transform mHealth data into digital biomarkers for disease detection, monitoring, and prevention. From the input of sensor data to the development of statistical modeling, machine learning, and deep learning algorithms, the DBDP provides tools for each step of the digital biomarker discovery process (Fig. 1). The DBDP abides by the findable, accessible, interoperable, reusable (FAIR) guiding principles to make data and code Findable, Accessible, Interoperable, and Reusable [24]. The DBDP has already been used to support 10 studies with several more currently underway [15,25–28]. Currently, the DBDP supports the development of new digital biomarkers through a general pipeline with extensible modules consisting of preprocessing and exploratory data analysis (EDA) tools to make the development of new digital biomarkers standardized and replicable. Currently, DBDP modules calculate and utilize resting heart rate (RHR), glycemic variability, insulin sensitivity status, exercise response, inflammation, heart rate variability, activity, sleep, and circadian patterns to predict health outcomes, and then we plan to integrate new digital biomarker modules relating to BioMeT data harmonization, pre-processing, EDA, predictive model building, and cardiometabolic disease research into the DBDP. The DBDP is a resource for the digital biomarker community that provides open-source modules in order to widen the scope of digital biomarker validation with standard frameworks, reduce duplication by comparing the existing digital biomarkers, and stimulate innovation through community outreach and education.

## Methods

### Software Specifications

The DBDP is an open-source software resource published in GitHub with Apache 2.0 licensing. We have developed a Wiki page with a user guide explaining on how to use the DBDP. We also provided complete instructions for contributing to the DBDP. We have adopted the Contributor Covenant (v2.0) [29] code of conduct. Code packages and digital biomarker modules are developed using a variety of programming languages that are integrated through containerization. The software is required to have specific documentation in order to be adopted into the DBDP. The DBDP is available at dbdp.org.

### DBDP Landscape

**Fig. 2.**
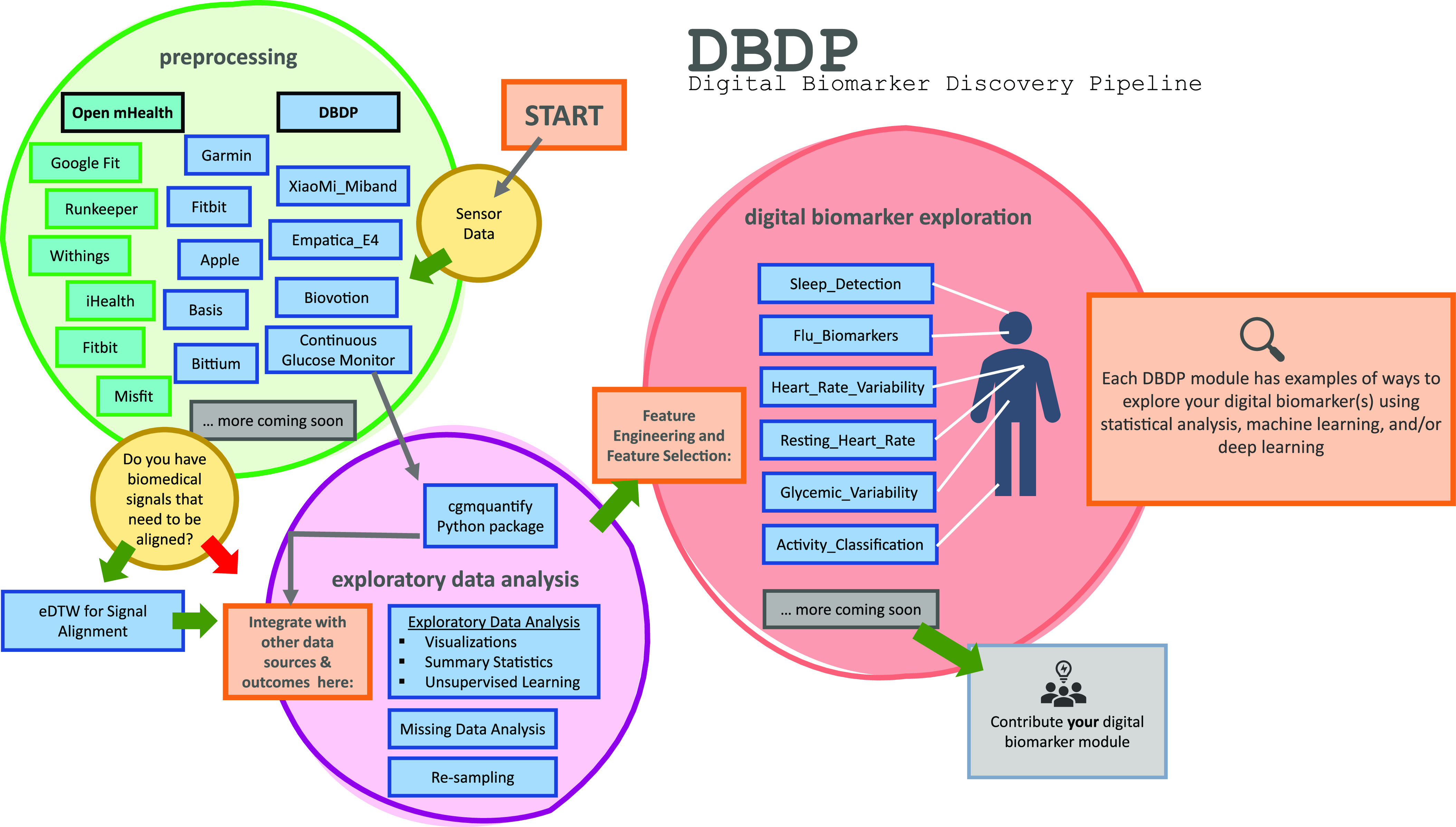
Current DBDP landscape.

DBDP modules have been developed to enable the discovery of new digital biomarkers and the comparison of the existing digital biomarkers. Additionally, the DBDP has been configured in a modular framework in order for code to be extensible to a variety of digital biomarker development studies and applications. Currently, DBDP modules calculate and utilize RHR, glycemic variability, insulin sensitivity status, exercise response, inflammation, heart rate variability, activity, sleep, and circadian patterns to predict health outcomes (Fig. 2) using statistics, data analytics, and machine learning algorithms such as regressions, random forests, and long-short-term memory models. The DBDP currently supports CGMs, ECG, and wearable watches Empatica E4, Garmin vivofit, and vivosmart, Apple Watch, Biovotion, Xiaomi Miband, and Fitbit. The EDA, RHR, heart rate variability, and glucose variability modules are currently device agnostic, and other modules are currently being configured to be device agnostic.

While there are many challenges to using ground truth measurements and they are not always available, particularly for new types of measurements, when they are available, they are necessary to verify and validate the digital biomarkers in the DBDP [8].

### Using the DBDP

The DBDP is intended to be used by researchers, clinicians, and anyone with an interest in exploring digital biomarkers. The DBDP is available as a general pipeline for wearables data pre-processing and conducting EDA with generic settings and recommendations for best practices. Individual modules can be tailored and combined to meet specific digital biomarker discovery applications and use cases. Additionally, users can submit a request for DBDP developers to build new biomarkers, features, or device pre-processing modules as detailed in the DBDP User Guide. The DBDP development team will work closely with users who have new devices to integrate into the DBDP pre-processing module. Detailed specifications for each algorithm and module are available in the DBDP to facilitate their use and adoption. The Digital Biomarker Development Resources Guide in the DBDP includes resources on choosing a wearable sensor, data handling, validation of data, and digital medicine in general.

### Contributing to the DBDP

In order to contribute to the DBDP, there are requirements for formatting and documentation that are available in our Contributing Guidelines. DBDP modules and algorithms are not automatically accepted and are subject to a rigorous review by the DBDP development team to ensure that the algorithm functions as documented. When contributing to the DBDP, an “Issue” is created. DBDP development team members will be assigned to the issue and will review it. In this way, the original developer will be openly reviewed and will be required to make any changes to their software before being accepted into the DBDP.

## Results

The DBDP provides generic and adaptable modules for pre-processing data and conducting EDA. The goal of the general pipeline is to allow for new digital biomarker development through a modular framework with tools for pre-processing and EDA that are extensible to a variety of digital biomarkers. Additionally, the DBDP contains multiple modules for specific digital biomarkers and research use cases (Fig. 2). These specific modules can be adapted to new digital biomarker research or can be used to compare new digital biomarkers to the pre-existing digital biomarkers. This enables researchers to compare their own digital biomarkers with pre-existing digital biomarkers, reducing duplication and stimulating innovation. We plan to further validate these modules and provide the module and a reference dataset as a resource to the community for digital biomarker benchmarking. Below, we will explore the general pipeline and three specific digital biomarkers included in the DBDP. We have chosen these specific modules because they showcase the diversity of the DBDP modules and provide end-to-end solutions that have been utilized in a number of projects. Additionally, they highlight the performance of the pipeline with different devices and use cases.

### General DBDP

The goal of the general DBDP is to provide a set of extensible tools for the development of new digital biomarkers. This general framework provides a standard framework for pre-processing and exploring data for digital biomarker development.

Our pre-processing module is developed to be device agnostic. We have processing and signal alignment modules for numerous wearable sensors. When comparing multimodal signals, signals that have different resolutions, or sampling frequencies can be difficult to align. Our EventDTW method allows for this signal alignment in our pre-processing module [29].

**Fig. 3.**
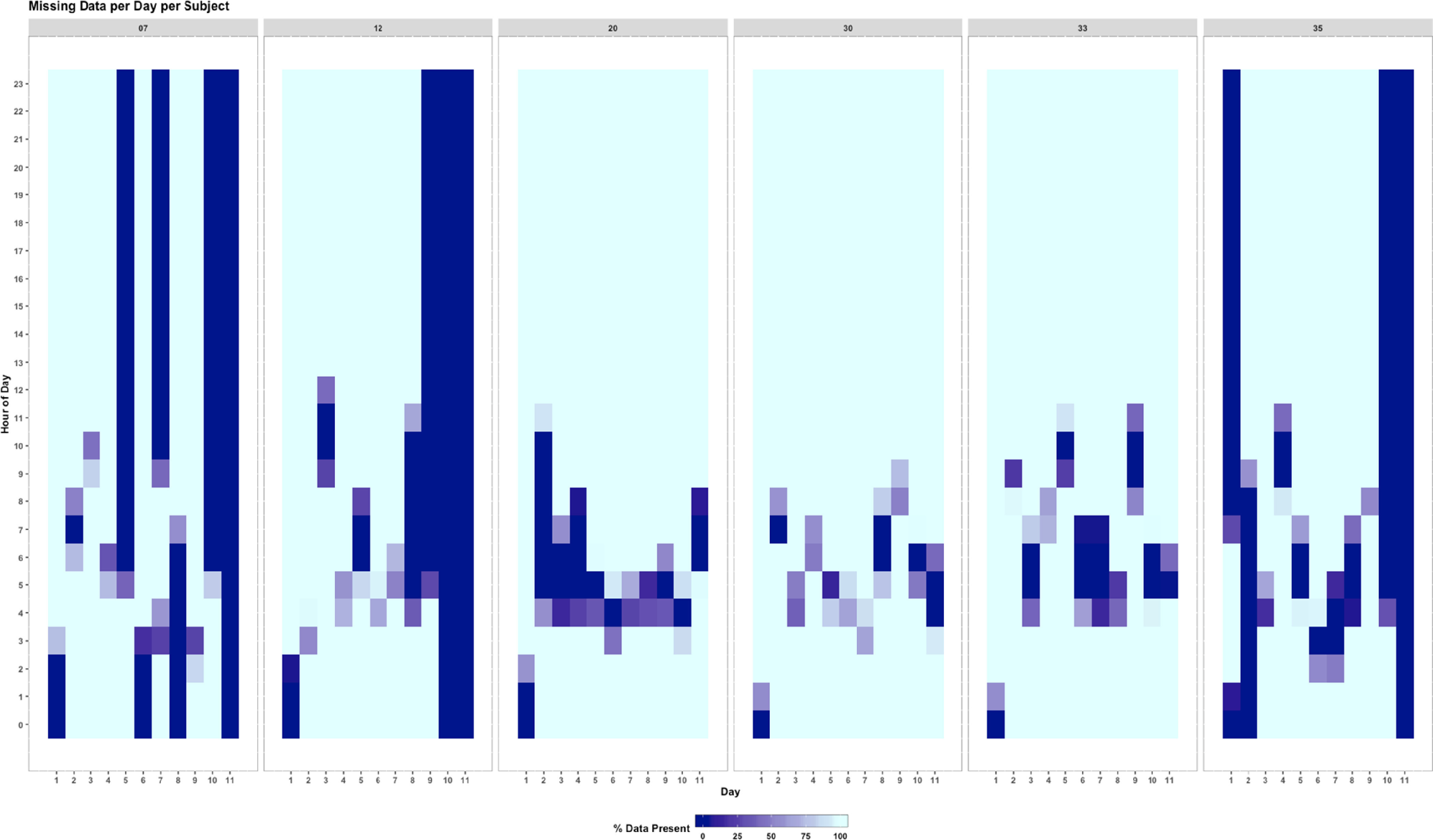
Missing data visualization available in the DBDP EDA module. This figure shows the percent of wearable data present per day and per hour for six study participants during a 10-day influenza exposure study [25].

EDA is an important step in the pipeline of digital biomarker development. EDA can uncover structure and trends in large mHealth datasets, including outliers, missingness [25], and relationships between variables, and can be helpful to visualize the data (e.g., Fig. 3) [25]. EDA is not a strictly defined process, and therefore resources are often sporadic. Our goal with the EDA module is to pool the common mHealth EDA methods that we and others use into one cohesive architecture, and to iterate over them to optimize and standardize mHealth EDA. Our primary goal with the EDA module is to standardize the process of EDA to enable structured mHealth data exploration.

### DBDP Module 1: RHR

**Fig. 4.**
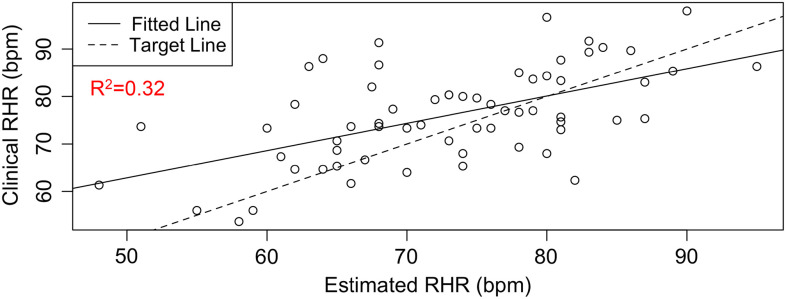
RHR module available in the DBDP. Clinical validation of our RHR algorithm against clinical data [8,30].

RHR characterizes several health conditions, such as type 2 diabetes (T2D) and cardiovascular diseases. The DBDP provides a personalized, accessible, and transparent method for estimating RHR from photoplethysmography-based wearable devices (e.g., Fitbit) data. To date, there has not been a comprehensive evaluation of how to best characterize RHR, which varies differently over time and between individuals with different activities and rest habits. Current methods for obtaining RHR require hands-on clinical measurements or utilize proprietary methods based on wearable device data. We have developed this module in order to increase the accessibility and transparency of RHR as a digital biomarker and to move toward a standardized and consistent RHR calculation method. This novel estimation model [30] (1) considers an individual’s heart rate data, (2) finds the subset of low exercise/step intensity heart rate data corresponding to a minimal rolling sum of steps (*n*) within a window size of *m* minutes, (3) searches for optimal values of parameters *n* and *m* by minimizing a standard deviation penalty function which quantifies the difference in variation between the distributions, and (4) outputs the RHR estimate as the median of the optimal low exercise/step intensity subset of personal heart rate data. This method has been validated against RHR clinical data through the STRONG-D study using Fitbits for type II diabetic adults (Fig. 4) [31]. This method is currently also being validated on infectious disease data collected using the Empatica E4 wearable wristband [32]. The RHR module is our first step toward developing a community standard RHR calculation method for digital biomarker development. It showcases the RHR metric, which is commonly used but is calculated in a variety of different ways. Within the DBDP, RHR definitions can be iterated over by the community, bringing together diverse needs to develop a robust and well-accepted method for RHR calculation in digital biomarkers.

### DBDP Module 2: Sleep and Circadian Rhythms

**Fig. 5.**
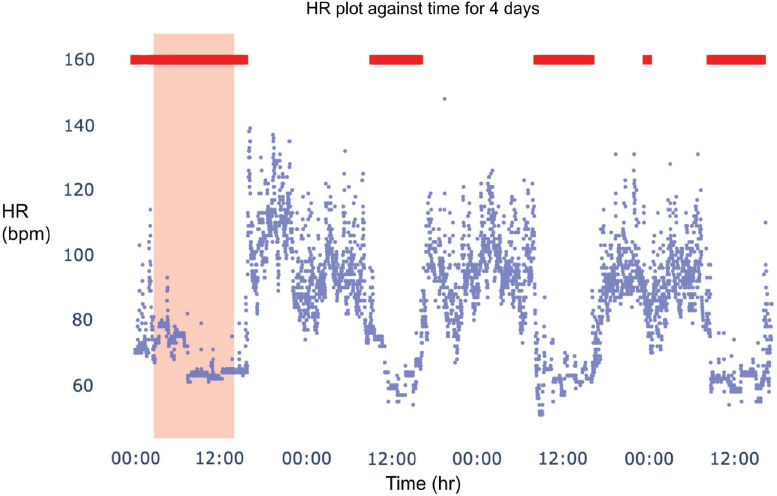
Sleep detection and disruption module available in the DBDP. Validation of sleep detection algorithm. Blue dots denote heart rate values at a point in time. Orange-shaded area is the reported sleep period by the proprietary commercial algorithm from the device manufacturer, and red rectangles indicate periods of sleep detected using this module.

Accurate sleep detection is necessary to determine the circadian rhythm and to discover relationships between circadian rhythm and sleep and wake characteristics. Currently, many sleep detection algorithms are available to consumers depending on either manual entry of sleep times or assumed sleep clock schedules. Such algorithms would fail to detect abnormal sleep times, especially for shift workers. The sleep detection algorithm in this DBDP module is developed to address this and expand on the applicability of currently available algorithms. This algorithm (1) identifies likely sleep and wake periods, (2) trains several different models to predict sleep and wake periods based on heart rate and activity measurements, and (3) chooses a method, that is, logistic regression, support vector machine to assign sleep/wake labels to periods [26]. This algorithm has been validated in wearable data from shift workers against participant-recorded sleep schedules and wearable-reported sleep times (Fig. 5). This module was developed because the commercial sleep detection algorithm does not accurately detect when the participant recorded that they were sleeping but the DBDP algorithm, based on heart rate and activity measurements, does accurately detect sleep. The Sleep and Circadian Rhythms module showcases an end-to-end solution that improves upon currently existing sleep detection algorithms [33,34]. We have included this module to show an end-to-end solution that is evolving as researchers contribute their findings.

### DBDP Module 3: cgmquantify for CGM Data

**Fig. 6.**
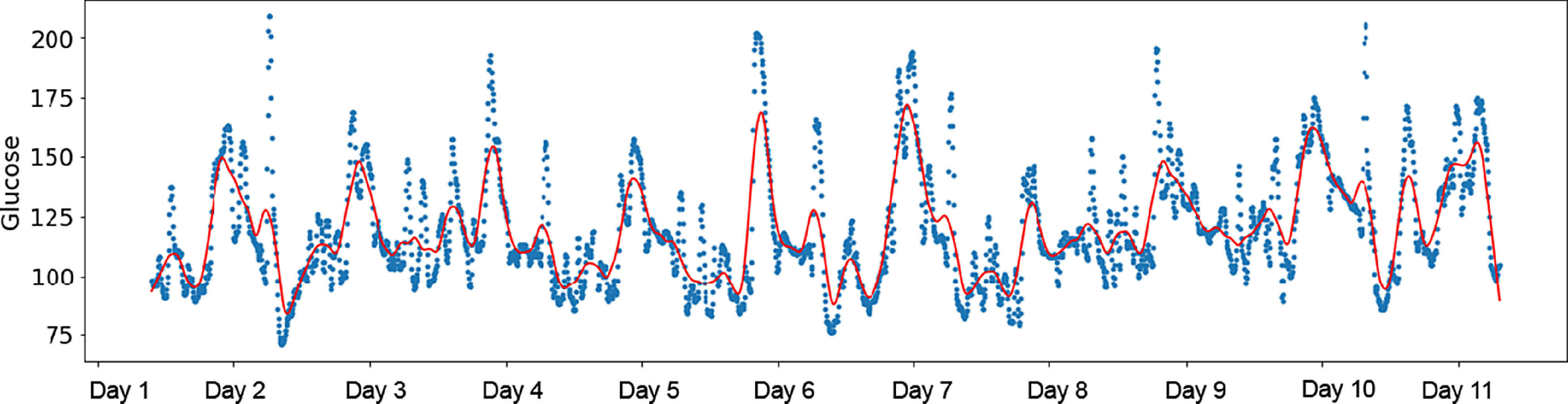
cgmquantify Python package available in the DBDP. Example of an LOWESS-smoothed visualization created using the cgmquantify package. LOWESS, locally weighted scatterplot smoothing.

Glucose and glycemic variability are indicative of hyperglycemia, hypoglycemia, and risk for developing prediabetes andT2D. They are also indicators of glycemic control, which is an important metric when evaluating the health of both type 1 diabetes and T2D patients. To our knowledge, there are currently no existing open-source software modules in Python that calculate these metrics from interstitial CGMs. The cgmquantify package we have developed includes 25+ summary metrics of glucose and glycemic variability [28]. The cgmquantify package also provides a comprehensive set of visualizations and statistical analyses to examine CGM data over time (individual) and provides resources for longitudinal studies with CGM (Fig. 6). As more researchers and clinicians begin utilizing CGM data to answer questions relating to prediabetes, T1D, and T2D, the need for a validated, standardized resource is necessary. As we have seen with the Open APS community, analysis of CGM data is not limited to researchers and clinicians but includes patients themselves [35]. By providing the DBDP cgmquantify module as an open-source resource, we hope to encourage patients to interact with their own data, determine personalized insights, and make meaningful contributions to the digital health landscape. Cgmquantify is a comprehensive function-based software tool that demonstrates how a deployed module from the DBDP may be used and recycled across a variety of research aims. Cgmquantify is a published Python package that has been developed as an MD2K Cerebral Cortex algorithm [28].

### DBDP for Education

The DBDP is currently being utilized for education on digital health and to spread community awareness on the digital biomarker discovery process. As a part of the Duke University Biomedical Engineering and Biostatistics and Bioinformatics undergraduate and graduate curriculums, the DBDP is being employed for students to learn and expand upon existing mHealth data science projects. The DBDP is also being utilized in the Rhodes Information Initiative Data+ Summer Research program for undergraduate students in a project on human activity recognition. One of the primary goals of this project is for students to develop their own modules within the DBDP. In the upcoming conferences, including the Women in Data Science Conference, we will be presenting DBDP workshops and tutorials. Based on our experiences using the DBDP for education, we have put together a Wiki with tutorials on how to adapt the DBDP in a variety of educational settings, including classrooms, workshops, case studies, and student projects. The DBDP is being utilized widely for multidisciplinary education, and we encourage its use for educational purposes in addition to research and development.

## Discussion

The growth in BioMeTs, including mHealth and wearables, has driven a surge in digital biomarker research. However, the lack of standardization and open-source computational tools has limited collaborative research and has resulted in numerous digital biomarkers in use that have not been validated [8,15]. Proprietary algorithms from wearable manufacturers do not provide information on their verification and validation processes, making it difficult to confirm the validity of aggregate metrics and digital biomarkers [8,36]. Additionally, this limits the accessibility of digital biomarker discovery research to only researchers with strong computational skills which may prevent experts with relevant domain knowledge from entering the field. Creating the DBDP, an open-source software platform for collaborative efforts in the digital biomarker space, with detailed documentation and user training, supports exploration and validation of digital biomarkers and enables multidisciplinary collaboration.

Much like in the field of genomics two decades ago, the mHealth technology renaissance has produced rapid expansion in digital health data. In order to balance this growth with an appropriate level of evaluation, we must develop standards and open-source resources to encourage the continued expansion of digital biomarker discovery like the open-source infrastructure driving genomics research [21–23]. The DBDP is an open-source software tool for making sense of data derived from BioMeTs and for discovering digital biomarkers from mHealth and wearables data. The DBDP currently enables the development of new digital biomarkers and the comparison of existing digital biomarkers. We are currently working toward rigorous validation of DBDP modules in order to provide them as a resource to the community for digital biomarker benchmarking.

In order to increase the reach of the DBDP and enable collaborations across the field of digital biomarker research, we plan to integrate the DBDP with the Open mHealth interoperable data collection architecture. Open mHealth has a large community with over 6500 developers. The Open mHealth tools for data collection are standards in industry [37]. Open mHealth is focused on collecting and storing wearable and mHealth data based on an open reference standard that is currently under ballot to become an official IEEE global standard [38]. Open mHealth tools include Shimmer, a data aggregator for mobile sensors (i.e., iHealth, GoogleFit). While Open mHealth’s syntactic and semantic standards facilitate interoperability, they do not address the clinical validity of the represented biomarker. To address clinical validity, we aim to integrate the Open mHealth framework into the DBDP. Specifically, we are currently creating a pipeline to automatically format the JSON outputs of Open mHealth’s Shimmer tool to function as direct inputs into the DBDP. In addition to outreach and education, we plan to integrate 100 new digital biomarker modules relating to BioMeT data harmonization, pre-processing, EDA, predictive model building, and cardiometabolic disease research into the DBDP by the end of 2022.

We also encourage the digital biomarker community to contribute open-access code, algorithms, and benchmarking datasets to the DBDP following the FAIR principles. In order to facilitate the verification and validation, and continued exploration of digital biomarker applications, the DBDP provides resources, a platform, and an online community to make this a reality. We envision the DBDP becoming a standard for the digital biomarker community and an epicenter of digital health collaboration and exploration.

## Code Availability

The DBDP is available at http://dbdp.org. The DBDP is open source, licensed with Apache 2.0, with no restrictions and is available at https://github.com/Big-Ideas-Lab/DBDP. Contributions must follow the Contributor Covenant v2 code of conduct.

## Supplementary material

For supplementary material accompanying this paper visit https://doi.org/10.1017/cts.2020.511. click here to view supplementary material
