# Supplementary figures and images for: The digital biomarker discovery pipeline: An open-source software platform for the development of digital biomarkers using mHealth and wearables data
Source: J Clin Transl Sci. 2020 Jul 14;5(1):e19. doi: 10.1017/cts.2020.511 (PMC8057397; doi:10.1017/cts.2020.511)

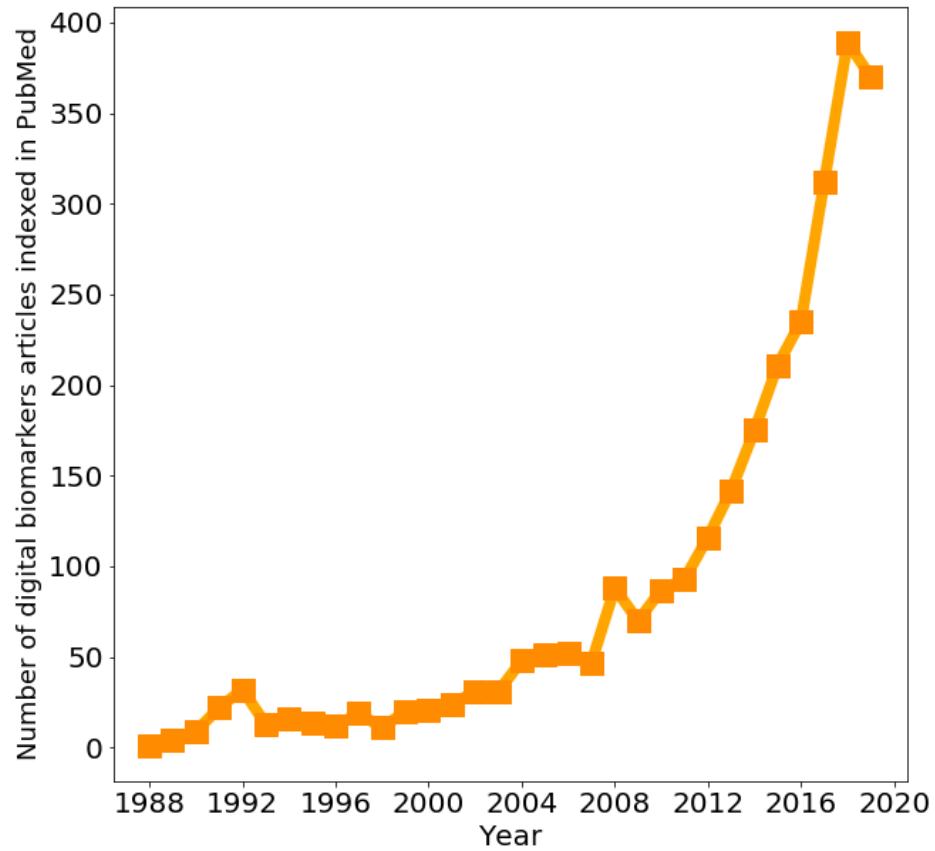

**Supplementary Figure 1.** Growth of digital biomarkers research studies indexed in PubMed.

Supplement: Supplementary file 1 [file S2059866120005117sup001.pdf]
